# Salivary biomarker profiling in prediabetes-associated periodontitis: role of adiponectin, resistin, and total matrix metalloproteinase-8

**DOI:** 10.3389/fdmed.2026.1873996

**Published:** 2026-06-19

**Authors:** Julie Toby Thomas, Betsy Joseph, Sajit Varghese, Nebu George Thomas, Kavya S, Toby Thomas, Timo Sorsa, Sukumaran Anil, Tuomas Waltimo

**Affiliations:** 1 Department of Oral and Maxillofacial Diseases, Helsinki University and University Hospital, Helsinki, Finland; 2 Department of Periodontics, Saveetha Dental College and Hospitals, Saveetha Institute of Medical and Technical Sciences, Chennai, India; 3 Department of General Medicine, Pushpagiri Institute of Medical Sciences and Research Centre, Thiruvalla, Kerala, India; 4 Department of Periodontics, Pushpagiri College of Dental Sciences, Thiruvalla, Kerala, India; 5 Department of Restorative Dentistry, College of Dentistry, Majmaah University, Al-Majmaah, Saudi Arabia; 6 Division of Periodontology, Department of Dental Medicine, Karolinska Institutet, Stockholm, Sweden; 7 Faculty of Dentistry, Chulalongkorn University, Bangkok, Thailand; 8 Global Research Cell, Dr. D. Y. Patil Dental College and Hospital, Dr. D. Y. Patil Vidyapeeth, Pimpri, Pune, India; 9 Department of Medicine, University of Basel, Basel, Switzerland

**Keywords:** adipokines, adiponectin, enzyme-linked immunosorbent assay (ELISA), periodontitis, prediabetes, resistin, salivary matrix metalloproteinases (MMP-8)

## Abstract

**Aim:**

This study evaluated the diagnostic potential of salivary total matrix metalloproteinase-8 (tMMP-8), adiponectin, and resistin in prediabetic adults with and without periodontitis and examined their associations with systemic and periodontal parameters.

**Materials and methods:**

Systemically healthy and prediabetic adults, categorized by periodontal status, underwent clinical, anthropometric, and metabolic assessments. A total of 84 adults aged 25–55 years were enrolled and stratified into four groups: prediabetes with periodontitis (PreDM-PD, *n* = 24), prediabetes with periodontal health (PreDM-PH, *n* = 19), systemically healthy with periodontitis (SH-PD, *n* = 22), and systemically and periodontally healthy controls (SH-PH, *n* = 19). Unstimulated saliva was collected to quantify tMMP-8, adiponectin, and resistin by sandwich ELISA. Statistical analyses included group comparisons (one-way ANOVA with Bonferroni *post-hoc* correction; Kruskal–Wallis with Dunn’s test for non-normally distributed variables), correlation analysis of biomarkers with systemic and periodontal measures, and principal component analysis (PCA) to define a composite inflammatory profile.

**Results:**

Participants with periodontitis had significantly higher periodontal parameters (*p* < 0.001). Salivary tMMP-8 was elevated in prediabetic individuals with periodontitis compared to other groups (*p* < 0.001), whereas adiponectin was lower in prediabetic groups versus controls (*p* < 0.01). tMMP-8 correlated positively with BMI, HbA1c, probing pocket depth (PPD), and clinical attachment loss (CAL). Adiponectin inversely correlated with HbA1c, PPD, CAL, and bleeding on probing. Resistin showed a weak positive correlation with CAL. PCA revealed an inflammatory profile (high tMMP-8, low adiponectin) explaining 37.1% of variance (KMO = 0.58; Bartlett’s *χ*² = 22.86, *p* < 0.001), effectively distinguishing prediabetic with periodontitis from healthy controls (AUC = 0.925, *p* < 0.001; sensitivity 87.5%, specificity 89.5%, Youden J = 0.77) and showing acceptable accuracy in periodontal health. Between-group differences for tMMP-8, adiponectin, PPD, CAL and BoP remained statistically significant after adjustment for age (ANCOVA, all *p* < 0.01).

**Conclusion:**

Salivary tMMP-8 and adiponectin composite profiles outperform single markers, supporting their use in non-invasive periodontal risk screening in metabolically vulnerable adults. Elevated tMMP-8 links periodontal and metabolic inflammation in prediabetes.

## 1 Introduction

Prediabetes, a metabolic state with elevated blood glucose below diabetes diagnosis levels, affects nearly one-third of U.S. adults (1). Individuals are often asymptomatic but face increased risk of progression to type 2 diabetes mellitus (T2DM) and related complications (2). Globally, diabetes is projected to rise 46% by 2050, reaching 853 million adults, posing a major health challenge (3). Periodontitis, a chronic inflammatory disease triggered by microbial imbalance and immune response, promotes insulin resistance through sustained inflammation and oxidative stress.

Pro-inflammatory cytokines, including interleukin-1 beta (IL-1β), tumor necrosis factor-alpha (TNF-α), and interleukin-6, together with excessive production of reactive oxygen species, interfere with insulin receptor signaling and impair glucose uptake at the cellular level (4). In hyperglycemia, oxidative stress and advanced glycation end-products increase MMP-8 release from neutrophils, which degrade insulin receptors and contribute to insulin resistance (5, 6). Elevated matrix metalloproteinase-8 (MMP-8) also accelerates tissue breakdown and triggers a proteolytic, pro-oxidative cascade marked by increased inflammatory biomarkers correlating with periodontal severity (7, 8). Recent systematic review has further reinforced that the collagenolytic activity of MMP-8 represents a unifying mechanistic link between dysglycemic stress and progressive periodontal tissue destruction (9).

Periodontal infection links to broader metabolic effects such as insulin resistance, pancreatic dysfunction, tissue apoptosis, and atherosclerosis (10). Elevated glycated hemoglobin (HbA1c) in prediabetics correlates with increased periodontal destruction, emphasizing the need for glycemic control to prevent irreversible damage (11). However, HbA1c alone may not reliably detect early prediabetes, highlighting the need for additional biomarkers (12). Prediabetes is 1.47 times more common in adults with moderate to severe periodontitis (13). Clinical attachment loss (CAL), a marker of periodontitis severity, increases the risk of prediabetes or diabetes by 1.1 times per unit increase in CAL >3 mm (14). However, prediabetic individuals may exhibit periodontal conditions similar to non-diabetics, reflecting the variable nature of this association (15). In Indian populations, in which the present study was conducted, the ICMR-INDIAB national survey has documented a particularly high burden of dysglycemia, with prediabetes prevalence estimated at 15.3% in adults, underscoring the public-health relevance of early detection strategies in this setting (16).

The underlying pathophysiological mechanisms linking these two conditions include shared phenotypic traits, such as increased body mass index (BMI), advancing age, a sedentary lifestyle characterized by prolonged physical inactivity, genetic predisposition (17), and elevated inflammatory markers. Increasing evidence further suggests that adipose tissue–derived cytokines (adipokines) play an important mechanistic role in the bidirectional relationship between periodontal and metabolic diseases. Adipokines such as adiponectin, which has anti-inflammatory and insulin-sensitizing effects, and resistin, which promotes inflammation and insulin resistance, may mediate this bidirectional link (18). Previous studies have demonstrated elevated resistin levels in periodontal inflammation and metabolic dysregulation, although findings regarding adiponectin remain inconsistent, potentially due to differences in assay methodologies and the inability to distinguish biologically active isoforms (19). In particular, the high-molecular-weight (HMW) isoform of adiponectin appears to be the most biologically active fraction with respect to insulin sensitization and anti-inflammatory signaling, and recent reviews have called for isoform-specific assays in periodontal-metabolic research (20). Salivary adiponectin may reflect oral inflammatory and immunometabolic interactions rather than systemic adiposity alone. Therefore, in prediabetes-associated periodontitis, adiponectin may gain diagnostic relevance when evaluated as part of a multi-biomarker inflammatory panel rather than as an isolated marker (21). Both salivary adiponectin and resistin correlate with serum levels, offering non-invasive biomarker options, though evidence in prediabetes is limited (22).

Saliva as a diagnostic medium provides real-time insight into periodontal inflammation, with MMP-8, especially its active form, strongly associated with tissue destruction in periodontitis and elevated in prediabetic individuals (23–25). Expert consensus and accumulating clinical evidence indicate that aMMP-8 demonstrates superior diagnostic performance compared with total MMP-8 in identifying active periodontal inflammation and grading disease severity (26, 27). Point-of-care aMMP-8 tests offer rapid, accurate chairside screening for periodontal inflammation, aiding early detection and monitoring among undiagnosed prediabetes or T2DM, supporting the growing concept of dental settings as potential sites for systemic disease screening and early risk identification (28). Multi-marker salivary biomarker panels, evaluated published between 2022 and 2024, have shown improved diagnostic accuracy compared with single-analyte approaches for both periodontal and metabolic risk stratification (29).

Based on this background, the present study aimed to evaluate and compare anthropometric, glycemic, periodontal, and salivary biomarker profiles among periodontitis patients with and without prediabetes and healthy controls. Additionally, the discriminative ability of salivary tMMP-8, adiponectin, and resistin was assessed, and a composite inflammatory profile score was developed by integrating biomarker levels to distinguish individuals with periodontitis and prediabetes, prediabetes without periodontitis, and systemically healthy periodontitis from systemically and periodontally healthy controls. This integrated biomarker approach may contribute to improving non-invasive identification of prediabetes-related inflammatory burden within dental care settings.

## 2 Materials and methods

### 2.1 Study design and participants

This cross-sectional study was conducted between 15 October 2024 and 15 January 2025 in the Department of Periodontology, Pushpagiri Institute of Medical Sciences and Research Center, Kerala, India. Ethical approval for this study was obtained from the Institutional Ethics Committee of Pushpagiri Institute of Medical Sciences and Research Centre, Kerala, India (IRB Reference No. 20/0112023). All participants provided written informed consent prior to participation. The study adhered to the principles of the revised Declaration of Helsinki (2013) and is reported in accordance with the STROBE guideline for cross-sectional studies.

Eighty-four participants aged 25–55 years were enrolled. The 25–55-year age window was chosen on biological and methodological grounds: the lower limit minimizes inclusion of aggressive forms of periodontitis that are more frequent in early adulthood, while the upper limit reduces confounding by age-related comorbidities, polypharmacy, and senescence-associated changes in adipokine biology, and corresponds to the demographic window in which prediabetes can be reliably identified before transitioning to overt T2DM. The participants were selected using a systematic random sampling method, whereby every 5th patient attending the clinic was invited to participate. The study included participants with periodontitis or those who were periodontally healthy and had prediabetes with HbA1c levels between 5.7% and 6.4%. To ensure accurate glycemic classification, only participants with no prior history of diabetes mellitus and not receiving any hypoglycemic medications were included. Participants were categorized as systemically healthy if they had HbA1c < 5.7% and no history of systemic disease.

**Figure 1:**
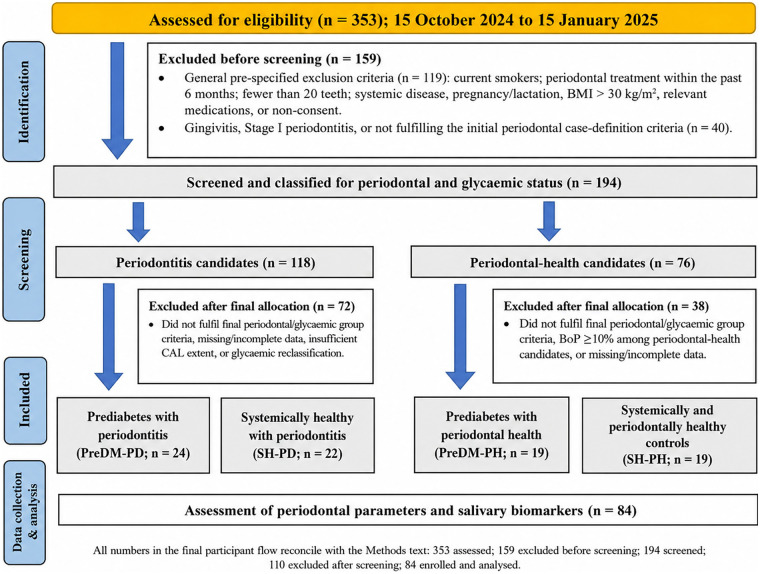
STROBE participant-flow diagram.

Accordingly, the sample comprised systemically healthy individuals with periodontitis, periodontally healthy individuals with prediabetes, and systemically and periodontally healthy controls. Of 353 individuals assessed for eligibility, 159 did not meet the initial inclusion criteria, 110 were further excluded for not fulfilling the case definitions of periodontitis or periodontal health (localized CAL not meeting case-definition criteria) or for other pre-specified exclusion criteria, resulting in a final study population of 84 individuals (Figure 1).

Participants were excluded if they had a history of systemic diseases (including T1DM or T2DM, hypertension, cardiovascular, hepatic, renal, malignant, bone, or autoimmune disorders), were pregnant or lactating, used medications affecting periodontal or metabolic status (NSAIDs, antibiotics within the past 6 months, or immunosuppressants), had received periodontal therapy within the past 6 months, had fewer than 20 teeth, used removable or fixed dentures or orthodontic appliances, or had a BMI >30 kg/m² or those who did not provide consent to participate in the study.

### 2.2 Diagnostic criteria for patient enrollment

Periodontitis was diagnosed and staged/graded according to the 2018 EFP/AAP classification (30, 31): interdental or buccal/oral CAL ≥ 3 mm at ≥ 2 non-adjacent sites, periodontal probing depth (PPD) ≥ 4 mm, and radiographic bone loss extending to the coronal or middle third of the root in > 30% of sites. Both stage and grade were assigned for every case. Periodontal grading was determined independently according to the 2018 EFP/AAP classification using the radiographic bone loss-to-age ratio as an indicator of progression rate. Accordingly, cases were classified across Stage II–III with Grade B predominance. The stage distribution per group is reported in Section 3.2. Exclusion of non-periodontal causes of attachment loss (e.g., gingival recession unrelated to periodontitis, distomolar pockets, endodontic lesions, vertical root fractures, or caries) was ensured. Periodontal health was defined as PPD ≤ 3 mm, bleeding on probing (BoP) < 10%, and absence of radiographic bone loss (32).

Clinical evaluation and evaluation of alveolar bone loss using digital panoramas was done by a single calibrated examiner (JTT). Intra-examiner reliability was assessed in 10 participants at a one-week interval. Cohen’s *κ* for presence of pocket ≥ 4 mm and CAL ≥ 3 mm was 0.85 (95% CI 0.78–0.92).

Prediabetes was defined by HbA1c values between 5.7% and 6.4% and/or fasting blood glucose (FBG) levels of 100–125 mg/dL (5.6–6.9 mmol/L). HbA1c was measured by an NGSP-certified high-performance liquid chromatography method (Bio-Rad D-10™ Hemoglobin Testing System) at the Department of Biochemistry, Pushpagiri Institute of Medical Sciences and Research Center. Both HbA1c and FBG were assessed for all participants. Glycemic status was determined using either recent medical records (within 8 weeks prior to enrollment) or study-site biochemical measurements; when necessary, repeat measurements were performed to ensure completeness and standardization. In cases where HbA1c and FBG values were discordant, participants were classified as prediabetic if either parameter fell within the prediabetic range. Participants with HbA1c < 5.7% and no history of diabetes were classified as systemically healthy. Participants were categorized into four groups according to their periodontal and glycemic status (Figure 1):

Group 1: Periodontitis with prediabetes (PreDM-PD), *n* = 24
Group 2: Periodontally healthy with prediabetes (PreDM-PH), *n* = 19
Group 3: Periodontitis with systemic health (SH-PD), *n* = 22
Group 4: Periodontally and systemically healthy controls (SH-PH), *n* = 19

### 2.3 Sociodemographic, anthropometric, and serum parameters

Participant information on age, residence, medical and dental history, duration of diabetes, smoking habits, medications, and oral hygiene practices was obtained using a closed-ended questionnaire (Appendix 1)*.* Physical examination was performed by a calibrated investigator (B.J.), who recorded body weight (kg) with a mechanical scale (Prestige, HM007, India) and height (m) with a stadiometer. BMI was calculated as weight (kg)/height (m²). Prior to data collection, the examiner was calibrated using duplicate measurements on 10 volunteers; intra-examiner technical errors of measurement were < 0.3 kg for body weight and < 0.5 cm for height and waist circumference. Equipment was calibrated daily using standard reference weight, and all anthropometric measurements were taken duplicate in accordance with standardized WHO protocols; the mean of the two readings was used for analysis. Serum FBG and HbA1c levels were also recorded.

### 2.4 Periodontal clinical examination

Periodontal clinical examination included assessment of PPD, CAL, the percentage of sites with BoP, plaque index (PI), and the number of teeth lost due to periodontitis. PPD, CAL, and BoP were assessed at six sites per tooth, excluding third molars, using a manual Williams periodontal probe (Hu-Friedy, Chicago, IL, USA) (33). Plaque index (34) was estimated as a percentage to assess the amount of plaque deposit. Mean PPD and CAL were recorded for every participant.

### 2.5 Sampling of saliva and assessment of biomarkers

Saliva samples were collected one day after the periodontal examination. Participants provided 5 mL of unstimulated saliva between 8:00 and 10:00 AM by pooling it in the mouth and allowing it to drain into a sterile funnel and container while seated upright (35, 36). Samples were stored at −20 °C until analysis.

Biomarker concentrations were measured using sandwich ELISA (enzyme-linked immunosorbent assay). Thawed saliva was centrifuged at 3,500 rpm (1,000  × g; Kenley, London, UK; rotor radius 7 cm) for 10 min, and the supernatant was used. Commercial ELISA kits quantified Human total MMP-8 (IT1690; 0.156–10 ng/mL, sensitivity 0.094 ng/mL), Adiponectin (IT3675; 0.47–30 ng/mL, sensitivity 0.938 ng/mL), and Resistin (IT1715; 0.16–10 ng/mL, sensitivity 0.056 ng/mL) (Immunotag™, G-Biosciences, USA). These kits were originally validated for use in serum/plasma; application to whole unstimulated saliva is acknowledged as a methodological limitation in Section 4.4, and absolute concentration values should be interpreted as semi-quantitative, while between-group comparisons within a single matrix and assay batch remain internally valid. For each assay, 100 μL of standards, blanks, or samples were added to antibody-precoated wells and incubated at 37 °C for 80 min. Wells were washed three times with 200 μL of 1 ×  wash buffer, followed by incubation with 100 μL of biotinylated antibody for 50 min at 37 °C. After washing, 100 μL of Streptavidin-HRP was added for 50 min and washed again. TMB substrate (90 μL) was incubated for 20 min at 37 °C in the dark, followed by 50 μL of stop solution. Optical density was immediately read at 450 nm.

### 2.6 Statistical analysis

Data was analyzed using IBM SPSS Statistics for Windows, Version 25.0 (IBM Corp., Armonk, NY, USA). Statistical significance was set at *p* < 0.05. Normality was formally tested with the Shapiro–Wilk test. For normally distributed continuous variables, group comparisons used one-way ANOVA with Bonferroni *post-hoc* correction; for non-normally distributed variables (including adiponectin and resistin), the Kruskal–Wallis test with Dunn’s *post-hoc* correction was used as a sensitivity analysis, and median (interquartile range) is reported alongside mean ± SD. Chi-square tests were used for categorical data. To address the influence of age, which differed significantly between groups, analyses of covariance (ANCOVA) with age (and, in a secondary model, sex) as covariates were performed for the primary biomarker outcomes (tMMP-8, adiponectin, resistin) and for the key clinical periodontal parameters (PPD, CAL, BoP).

Principal component analysis (PCA) with Varimax rotation was applied to the salivary biomarker data, with suitability confirmed by the Kaiser–Meyer–Olkin (KMO) measure of sampling adequacy (KMO = 0.58) and Bartlett’s test of sphericity (*χ*² = 22.86, df = 3, *p* < 0.001). Components with eigenvalues > 1 were retained; factor scores were used for further analysis. PCA was preferred over a simple equal-weighted composite because it derives data-driven weights that maximize explained variance and accommodate the inverse adiponectin–MMP-8 relationship through signed loadings. A weighted z-score composite (MMP-8 z−adiponectin z) was computed as a sensitivity analysis and yielded results closely concordant with the PCA-derived score. ROC curves evaluated diagnostic accuracy; optimal cut-off values were determined by maximizing the Youden index (J = sensitivity + specificity−1), with corresponding sensitivity and specificity reported (Table 1). Sample size adequacy was confirmed by *post hoc* power calculations (> 95% for primary comparisons), with clinically meaningful effect sizes detected (Cohen’s d ≥ 0.5) and *η*² > 0.25 for major outcome variables. The sample size for evaluating the discriminatory ability of tMMP-8 to identify periodontitis was calculated based on an AUC of 0.80 compared to a null-hypothesis AUC of 0.5, with a two-sided alpha of 0.05% and 80% power, ensuring adequate power to detect significant diagnostic accuracy.

**Table 1:** Area under the curve (AUC) values for the inflammatory profile score across group comparisons.

Comparison	AUC	SE	*p*-value	95% CI	Cut-off	Sens (%)	Spec (%)	Youden J
PreDM-PD vs SH-PH	0.925	0.042	<0.001***	0.844–1.000	0.31	87.5	89.5	0.77
PreDM-PH vs SH-PH	0.715	0.087	0.024*	0.543–0.886	−0.18	68.4	73.7	0.42
SH-PD vs SH-PH	0.758	0.076	0.005**	0.609–0.908	−0.05	72.7	73.7	0.46

Cut-off values were determined by maximizing the Youden index (J = sensitivity + specificity−1) on the Inflammatory Profile Score (z-units).

*** *p* < 0.001.

** *p* < 0.01.

* *p* < 0.05.

## 3 Results

### 3.1 Demographic and anthropometric characteristics and glycemic parameters

Demographic characteristics, oral health habits, anthropometric measures, and glycemic parameters of the study participants across the four groups (PreDM-PD, PreDM-PH, SH-PD, SH-PH) are summarized in Supplementary Table S1. PreDM-PD and SH-PD participants were older and exhibited higher BMI, WC, FBS, and HbA1c than SH-PH controls (*p* < 0.001). Mean ages by group were: PreDM-PD 47.2 ± 6.8 yr; PreDM-PH 46.5 ± 7.1 yr; SH-PD 44.9 ± 7.6 yr; SH-PH 35.8 ± 6.4 yr; F(3,80) = 13.65, *p* < 0.001. Gender distribution did not differ significantly between groups (PreDM-PD 13 M/11 F; PreDM-PH 9 M/10 F; SH-PD 12 M/10 F; SH-PH 9 M/10 F; *χ*² = 0.93, *p* = 0.82). Daily flossing was slightly more in PreDM-PD participants, and bleeding on brushing occurred more frequently in PreDM-PD, PreDM-PH, and SH-PD groups compared to SH-PH participants.

### 3.2 Assessment of clinical periodontal parameters

Within the periodontitis groups, stage distribution was as follows: SH-PD: Stage II 64% (14/22), Stage III 36% (8/22); PreDM-PD: Stage II 58% (14/24), Stage III 42% (10/24). Based on radiographic bone loss-to-age ratio assessment, most periodontitis cases were consistent with Grade B; however, formal grading categorization and statistical analysis were not performed.

BoP was highest in the SH-PD group (51.32  ±  14.41%), followed by PreDM-PD (38.96  ±  16.10%), and lower in PreDM-PH (12.16  ±  8.60%) and SH-PH (7.37  ±  4.37%) groups (F(3,80) = 58.65, *p* < 0.001, *η*² = 0.69). PI also differed significantly (F(3,80) = 28.55, *p* < 0.001, *η*² = 0.52), with highest plaque accumulation in SH-PD (40.23  ±  15.45%) and PreDM-PD (28.00  ±  6.91%), while periodontal health groups showed lower levels (PreDM-PH: 18.42  ±  6.52%; SH-PH: 14.05  ±  5.61%).

PPD was greater in PreDM-PD (4.41 ± 1.15 mm) and SH-PD (4.84 ± 0.99 mm) than in PreDM-PH (2.63 ± 0.48 mm) and SH-PH (2.58 ± 0.59 mm) (F(3,80) = 35.79, *p* < 0.001, *η*² = 0.57). CAL was significantly higher in both periodontitis groups compared with the periodontally healthy groups (F(3,80) = 142.18, *p* < 0.001). Mean CAL values were 5.74 ± 1.51 mm in the PreDM-PD group and 5.84 ± 1.13 mm in the SH-PD group, with no statistically significant difference observed between these two periodontitis groups.

GR differed significantly (F(3,80) = 26.42, *p* < 0.001), with the highest values in PreDM-PD and SH-PD and the lowest in the healthy groups. Mean number of missing teeth also varied significantly (F(3,80) = 17.34, *p* < 0.001) with SH-PD participants experiencing more tooth loss (3.06 ± 1.61) than PreDM-PD (2.71 ± 1.48) and SH-PH (1.00 ± 0.00) groups (Table 2). Sensitivity analyses using the Kruskal–Wallis test for non-normally distributed variables (GR, missing teeth) confirmed the same between-group differences (all *p* < 0.001).

**Table 2:** Comparison of demographic, systemic, and clinical periodontal parameters of the study groups.

Variable	PreDM-PD (*n* = 24)	PreDM-PH (*n* = 19)	SH-PD (*n* = 22)	SH-PH (*n* = 19)	F-statistic	*p*-value
Age (years)	47.2 ± 6.8	46.5 ± 7.1	44.9 ± 7.6	35.8 ± 6.4	13.65	<0.001*
Sex (M/F)	13/11	9/10	12/10	9/10	*χ*² = 0.93	0.82
BMI (kg/m²)	27.4 ± 3.2ᵃ	23.1 ± 2.5ᵇ	23.1 ± 2.6ᵇ	24.2 ± 3.5ᵇ	10.26	<0.001*
WC (cm)	102.7 ± 10.0ᵃ	103.2 ± 15.3ᵃ	92.1 ± 4.8ᵇ	90.1 ± 4.8ᵇ	10.08	<0.001*
FBG (mg/dL)	130.1 ± 15.1ᵃ	124.2 ± 15.0ᵃ	98.9 ± 6.9ᵇ	85.3 ± 7.7ᶜ	62.39	<0.001*
HbA1c (%)	6.1 ± 0.3ᵃ	6.0 ± 0.3ᵃ	5.1 ± 0.4ᵇ	4.9 ± 0.3ᵇ	79.11	<0.001*
BoP (%)	39.0 ± 16.1ᵃ	12.16 ± 8.60ᵇ	51.3 ± 14.4ᶜ	7.4 ± 4.4ᵇᵈ	58.65	<0.001*
PI (%)	28.0 ± 6.9ᵃ	18.4 ± 6.5ᵇ	40.2 ± 15.5ᶜ	14.1 ± 5.6ᵇᵈ	28.55	<0.001*
PPD (mm)	4.4 ± 1.2ᵃ	2.6 ± 0.5ᵇ	4.8 ± 1.0ᵃᶜ	2.6 ± 0.6ᵇᵈ	35.79	<0.001*
GR (mm)	2.0 ± 1.0ᵃ	1.6 ± 0.9ᵇ	1.8 ± 0.6ᵃᶜ	0.0 ± 0.0ᵈ	26.42	<0.001*
CAL (mm)	5.7 ± 1.5ᵃ	0.0 ± 0.0ᵇ	5.8 ± 1.1ᵃᶜ	0.0 ± 0.0ᵇᵈ	142.18	<0.001*
Missing Teeth (n)	2.7 ± 1.5ᵃ	1.0 ± 0.0ᵃ	3.1 ± 1.6ᵇ	1.0 ± 0.0ᵃ	17.34	<0.001*

* Statistically significant at the 1% level (*p* < 0.01); one-way ANOVA was used for continuous variables to test differences across the four groups, and Bonferroni *post-hoc* correction was used for pairwise comparisons. Values with different superscripted letters indicate a statistically significant pairwise difference (*p* < 0.05) by Bonferroni-adjusted comparisons; values with the same superscript indicate no significant pairwise difference (*p* > 0.05). Corrected F-values are shown in blue for variables in which the originally reported F-statistic was a transcription error; the corresponding *p*-values were recomputed from the original SPSS output. For GR and missing teeth, Kruskal–Wallis confirmatory analyses yielded *p* < 0.001.

PreDM-PD, prediabetes with periodontitis; PreDM-PH, prediabetes with periodontal health; SH-PD, systemically healthy with periodontitis; SH-PH, systemically and periodontally healthy controls; BMI, body mass index; WC, waist circumference; FBG, fasting blood glucose; HbA1c, glycated hemoglobin; BoP, bleeding on probing; PI, plaque index; PPD, probing pocket depth; GR, gingival recession; CAL, clinical attachment loss.

### 3.3 Analysis of salivary biomarkers

Table 3 shows significant differences in salivary biomarker levels among groups. tMMP-8 levels differed significantly (F(3,80) = 20.42, *p* < 0.001, *η*² = 0.43), with PreDM-PD exhibiting markedly higher concentrations (87.13 ± 31.58 ng/mL) than PreDM-PH (48.61 ± 12.29 ng/mL), SH-PD (50.87 ± 12.63 ng/mL), and SH-PH (46.92 ± 11.76 ng/mL). Non-parametric confirmation: Kruskal–Wallis H(3) = 36.91, *p* < 0.001.

**Table 3:** Salivary biomarker analysis.

Biomarker	PreDM-PD (*n* = 24)	PreDM-PH (*n* = 19)	SH-PD (*n* = 22)	SH-PH (*n* = 19)	F	*p* (ANOVA/KW)	*η*²
tMMP-8 (ng/mL)	87.1 ± 31.6ᵃ	48.6 ± 12.3ᵇ	50.9 ± 12.6ᵇ	46.9 ± 11.8ᵇ	20.4	<0.001** (KW *p* < 0.001)	0.43 (Large)
tMMP-8 median [IQR]	82.5 [62.4–108.7]	47.9 [40.1–56.8]	50.2 [42.0–58.6]	45.5 [38.7–55.1]	—	H = 36.91, *p* < 0.001	—
Adiponectin (ng/mL)	2.6 ± 5.6ᵃ	2.0 ± 1.8ᵃ	3.1 ± 2.5ᵃ	8.6 ± 7.7ᵇ	6.9	<0.01* (KW *p* < 0.001)	0.21 (Medium)
Adiponectin median [IQR]	1.4 [0.8–2.6]	1.6 [0.9–2.7]	2.4 [1.5–4.1]	6.2 [3.7–11.4]	—	H = 18.62, *p* < 0.001	—
Resistin (ng/mL)	4.0 ± 2.6ᵃ	2.1 ± 1.6ᵇ	3.5 ± 2.2ᵃ	3.4 ± 2.8ᵃᵇ	2.4	NS (KW *p* = 0.091)	0.08 (Small)

One-way ANOVA with Bonferroni *post-hoc* correction was used for continuous variables; Kruskal–Wallis (KW) tests with Dunn’s *post-hoc* correction were performed as non-parametric sensitivity analyses, and median [IQR] is reported alongside mean ± SD where data were skewed. Values with different superscripted letters indicate significant pairwise difference (*p* < 0.05); the same superscript indicates no significant difference. Effect sizes: small (*η*² = 0.01), medium (*η*² = 0.06), large (*η*² ≥ 0.14).

PreDM-PD, prediabetes with periodontitis; PreDM-PH, prediabetes with periodontal health; SH-PD, systemically healthy with periodontitis; SH-PH, systemically and periodontally healthy controls; tMMP-8, total matrix metalloproteinase-8; IQR, interquartile range.

Statistically significant *p < 0.01

** *p* < 0.001; NS, not significant.

Adiponectin levels differed moderately (F(3,80) = 6.88, *p* < 0.01, *η*² = 0.21), with the control group showing the highest mean level (8.57 ± 7.73 ng/mL) and lower levels in the prediabetic groups (PreDM-PD: 2.62 ± 5.62 ng/mL; PreDM-PH: 1.96 ± 1.81 ng/mL). Because adiponectin distributions were skewed, median (IQR) values are also reported in Table 3, and a non-parametric sensitivity analysis (Kruskal–Wallis H(3) = 18.62, *p* < 0.001) confirmed the parametric finding.

Resistin levels showed minor differences, with the highest levels in PreDM-PD (4.00 ± 2.61 ng/mL), but did not reach statistical significance (F(3,80) = 2.42, *p* = 0.073, *η*² = 0.08; Kruskal–Wallis H(3) = 6.47, *p* = 0.091).

After adjusting for age (and additionally for sex) using ANCOVA, between-group differences in tMMP-8 (F(3,79) = 17.86, *p* < 0.001), adiponectin (F(3,79) = 5.93, *p* = 0.001), PPD, CAL and BoP remained statistically significant, indicating that the observed group differences are not solely driven by the age imbalance between the prediabetic/periodontitis groups and the healthy controls.

### 3.4 Associations of salivary biomarkers with systemic and periodontal parameters

**Figure 2:**
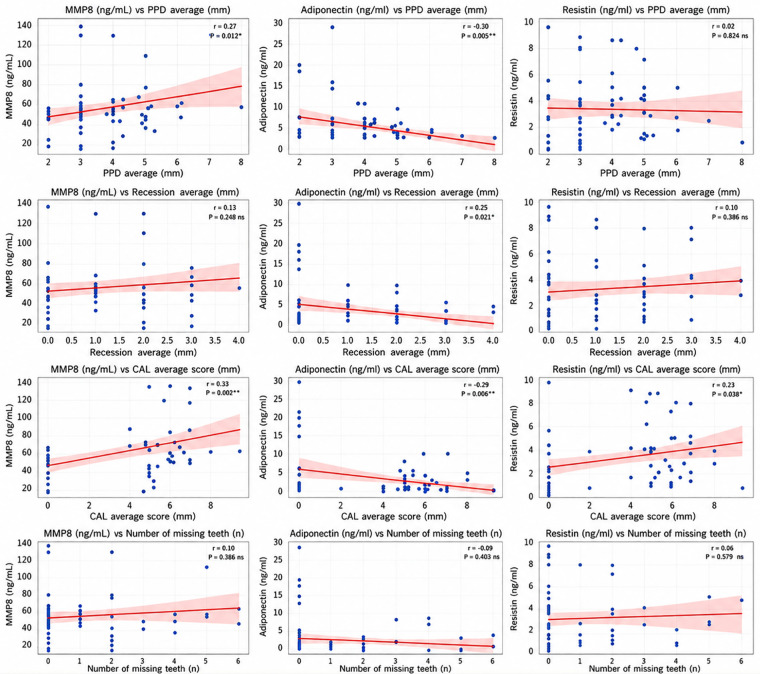
Scatter plots with linear regression lines showing associations between salivary biomarkers and clinical periodontal parameters. r values indicate Pearson correlation coefficients. **Statistically significant at the 1% level (*p* < 0.01); *significant at the 5% level (*p* < 0.05); ns, not significant.

Correlation analysis assessed relationships between systemic parameters, clinical periodontal variables, and salivary biomarkers (MMP-8, adiponectin, resistin). MMP-8 showed significant positive correlations with BMI (*r* = 0.402, *p* < 0.01), WC (*r* = 0.259, *p* < 0.05), FBS (*r* = 0.267, *p* < 0.05), HbA1c (*r* = 0.309, *p* < 0.01), PPD (*r* = 0.273, *p* < 0.05), and CAL (*r* = 0.332, *p* < 0.01), with weaker positive trends observed for weight and BoP (Supplementary Table S2*,* Figure 2). This indicates that elevated salivary MMP-8 reflects both systemic metabolic dysregulation and active periodontal destruction.

In contrast, adiponectin correlated negatively with WC (*r* = −0.259, *p* < 0.05), FBS (*r* = −0.464, *p* < 0.01), HbA1c (*r* = −0.494, *p* < 0.01), PPD (*r* = −0.298, *p* < 0.01), CAL (*r* = −0.292, *p* < 0.01), BoP (*r* = −0.259, *p* < 0.05), and PI (*r* = −0.222, *p* < 0.05), suggesting a protective role in glycemic control and periodontal health.

Resistin showed weaker correlations, with a significant positive association only with CAL (*r* = 0.226, *p* < 0.05), and no significant correlations with systemic metabolic or other periodontal parameters.

### 3.5 Principal component analysis and discriminative ability of the inflammatory profile score

Principal component analysis identified a single dominant component (PC; eigenvalue = 1.177) explaining 37.1% of the total variance among tMMP-8, adiponectin, and resistin. KMO sampling adequacy was 0.58 and Bartlett’s test of sphericity was significant (*χ*² = 22.86, df = 3, *p* < 0.001), supporting the suitability of the data for PCA. The component was primarily defined by a strong positive loading for tMMP-8 (0.861) and a moderate negative loading for adiponectin (−0.603), while resistin contributed minimally (0.085). Biomarker values were standardized to z-scores using the sample mean and standard deviation, and the Inflammatory Profile Score (IPS) was calculated as the weighted sum of standardized biomarker values using the PCA loadings. The component accounted for 77.1% of the variance in tMMP-8 and 61.3% in adiponectin and was used as a composite inflammatory score for further analysis (Supplementary Table S3).

**Figure 3:**
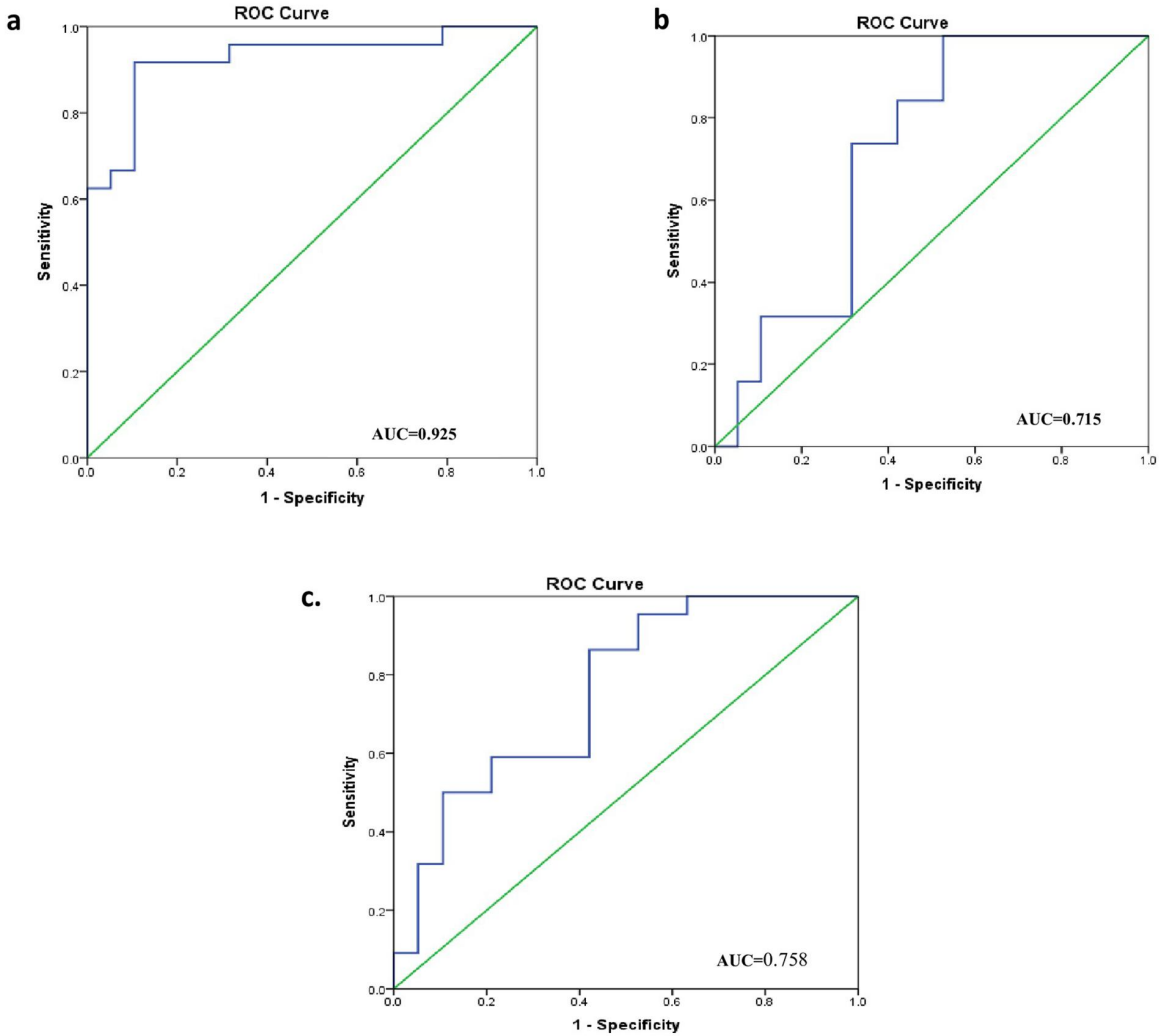
Receiver operating characteristic (ROC) curves for the inflammatory profile score across group comparisons: **(a)** PreDM-PD vs SH-PH; **(b)** PreDM-PH vs SH-PH; **(c)** SH-PD vs SH-PH.

The Inflammatory Profile Score effectively discriminated participant groups: excellent discrimination was observed between PreDM-PD and SH-PH (AUC = 0.925, SE = 0.042, *p* < 0.001, 95% CI 0.844–1.000; optimal cut-off 0.31; sensitivity 87.5%, specificity 89.5%, Youden J = 0.77). Discrimination was acceptable between PreDM-PH and SH-PH (AUC = 0.715, SE = 0.087, *p* = 0.024; cut-off −0.18; sensitivity 68.4%, specificity 73.7%, Youden J = 0.42), and between SH-PD and SH-PH (AUC = 0.758, SE = 0.076, *p* = 0.005; cut-off −0.05; sensitivity 72.7%, specificity 73.7%, Youden J = 0.46). The highest discriminatory accuracy was observed for the PreDM-PD group (Table 1, Figure 3).

## 4 Discussion

Prediabetes is increasingly recognized as a risk factor for periodontitis, with metabolic dysregulation promoting periodontal inflammation and tissue destruction even before diabetes onset (37). Salivary biomarkers provide a non-invasive approach to study this interplay. MMP–8, a neutrophil-derived collagenase, plays a central role in matrix degradation and periodontal tissue breakdown (23). Adiponectin exerts anti-inflammatory effects, whereas resistin is pro-inflammatory independent of obesity (18). This study assessed whether their salivary levels reflect systemic profiles by comparing salivary adiponectin, resistin, and tMMP–8 in prediabetic adults with and without periodontitis, aiming to evaluate their diagnostic potential for detecting periodontal inflammation in metabolically at-risk populations.

### 4.1 Anthropometric, systemic, and periodontal parameters

In this study, demographic confounders such as gender, smoking status, and oral hygiene habits were relatively similar across groups, minimizing their potential influence on the associations between prediabetic status and periodontal parameters. Although age differed between groups, ANCOVA confirmed that the principal biomarker and periodontal differences persisted after adjustment for age (and additionally for sex), supporting the robustness of the findings. Prediabetic participants, regardless of periodontal status, exhibited significantly higher FBS and HbA1c levels, along with greater BMI and WC, reflecting underlying metabolic dysregulation. These findings are consistent with previous reports showing that cumulative exposure to higher body weight and prolonged overweight status is associated with incident prediabetes, highlighting the role of excess adiposity and its duration in metabolic dysfunction (38).

Previous studies among prediabetic patients showed that poor glycemic control was significantly associated with increased periodontitis severity (39). In our study, SH-PD and PreDM-PD participants showed higher BoP, PI, PPD, CAL, GR, and tooth loss than controls. Notably, prediabetic individuals with periodontal health had elevated BoP despite similar CAL and PPD, suggesting increased inflammatory susceptibility linked to glycemic imbalance. Consistent with Altamash et al., no significant periodontal differences were observed between periodontitis patients with and without prediabetes, indicating similar periodontal conditions in prediabetic and non-diabetic subjects. Overall, these findings suggest that prediabetes contribute to systemic and localized inflammatory changes that may increase periodontal vulnerability (40).

### 4.2 Salivary biomarkers and metabolic-periodontal links

Unstimulated saliva was used to measure adiponectin, tMMP-8, and resistin due to its non-invasive nature and correlation with plasma biomarkers (41). Salivary tMMP-8 was significantly elevated in the prediabetic periodontitis group (87.1 ± 31.6 ng/mL) compared with other groups, reflecting periodontal inflammation exacerbated by metabolic dysregulation. Elevated tMMP-8 indicates subclinical inflammation and may serve as an early biomarker to prevent progression to T2DM (2). These findings align with Miller et al., who reported the highest salivary MMP-8 levels in diabetics with periodontitis compared with non-diabetic periodontitis and healthy controls, highlighting MMP-8’s strong association with periodontal severity and hyperglycemia (mean HbA1c 9.0 ± 1.87%), and suggesting that diabetes upregulates MMP-8 expression and activation (23).

Adiponectin levels were highest in healthy controls (8.57  ±  7.73 ng/mL) and lowest in prediabetic groups, supporting its protective role, consistent with decreased serum adiponectin in periodontitis patients (42). Resistin, a pro-inflammatory adipokine that promotes insulin resistance and upregulates cytokines such as TNF–*α* and IL–12, was significantly higher in the PreDM–PD group, indicating that periodontitis contributes to its elevation (43). These findings highlight the interplay between metabolic status and periodontal inflammation, with salivary tMMP–8 and adiponectin serving as robust biomarkers.

Salivary tMMP–8 correlated positively with BMI (*r* = 0.402), WC (*r* = 0.259), FBS (*r* = 0.267), and HbA1c (*r* = 0.309), reflecting its role as a marker of systemic metabolic dysregulation and aligning with its strong correlation with PPD ≥ 5 mm. In deepened periodontal pockets, MMP−8 is activated into collagenolytic active MMP–8, enhancing periodontal tissue destruction (23). Conversely, adiponectin showed negative correlations with WC (*r* = −0.259), FBS (*r* = −0.464), and HbA1c (*r* = −0.494), supporting its protective role in glycemic control. Elevated resistin and reduced adiponectin in periodontitis patients further link metabolic dysregulation to periodontal inflammation (44).

Total MMP−8 correlated positively with CAL (*r* = 0.332) and PPD (*r* = 0.273), reflecting its role in active periodontal tissue destruction (45, 46). This aligns with previous findings where salivary IL-1β, MMP-8, and resistin correlated with periodontal parameters, with MMP-8 showing the strongest association with PPD ≥ 5 mm in T2DM patients (23). Adiponectin showed negative correlations with BoP, PI, PPD, GR, and CAL (*r* = −0.222 to −0.298), supporting its anti-inflammatory and tissue-protective role. These results underscore the distinct contributions of salivary biomarkers in linking metabolic dysfunction to periodontal severity, consistent with prior reports where adipokines were influenced by BMI and gender but not directly by periodontal measures, likely due to prediabetes in this population (47).

Resistin showed weak correlations with clinical parameters, being significantly associated only with CAL (*r* = 0.226, *p* < 0.05), and not with systemic metabolic measures or other periodontal indices. Salivary resistin and other adipokines were not significantly altered in prediabetic participants, contrasting with elevated levels reported in T2DM (23, 48), likely reflecting the earlier metabolic stage in prediabetes with less pronounced inflammatory changes.

### 4.3 Composite salivary biomarker profiles for periodontitis detection in prediabetes

Principal component analysis identified a composite inflammatory signature with strong positive loadings for tMMP-8 and moderate negative loadings for adiponectin, explaining 37.1% of the variance, while resistin contributed minimally. PCA was preferred over a simple equal-weighted composite because it derives data-driven weights that maximize explained variance and accommodate the inverse adiponectin–MMP–8 relationship through signed loadings; a parallel sensitivity analysis using a weighted z-score composite (MMP-8 z – adiponectin z) yielded an AUC of 0.911 for PreDM-PD vs. SH-PH, closely matching the PCA-derived AUC of 0.925. These results align with prior studies showing MMP-8’s strong discriminatory ability to differentiate periodontitis from health (OR = 8.12) and from diabetes without periodontitis (OR = 5.09), highlighting its key role in periodontal disease detection and progression (23).

The Inflammatory Profile Score derived from this PCA showed strong discrimination for PreDM-PD versus SH-PH (AUC = 0.925, *p* < 0.001; sensitivity 87.5%, specificity 89.5%, Youden J = 0.77), highlighting salivary tMMP-8 and adiponectin as non-invasive markers of periodontal inflammation in metabolically at-risk populations. It also discriminated against PreDM-PH (AUC = 0.715, *p* = 0.024) and SH-PD (AUC = 0.758, *p* = 0.005) from controls, detecting early inflammatory changes. These results align with Gateva et al., who found lower serum adiponectin in prediabetes with good discriminative ability for insulin resistance (AUC = 0.728), while resistin and other adipokines showed no significant differences, supporting adiponectin as an early biomarker of metabolic dysfunction and periodontal risk (49).

In our study, biomarker combinations such as tMMP-8 with adiponectin outperformed single markers (AUC = 0.925). Integrating pro-inflammatory/collagenolytic (tMMP-8) and protective (adiponectin) salivary biomarkers via PCA can yield a biologically meaningful composite measure of periodontal and metabolic status, although its added diagnostic value should be weighed against simpler clinical screening approaches in terms of cost-effectiveness and practical implementation.

### 4.4 Limitations and future directions

A key limitation of this study is the relatively small sample size, which may have reduced statistical power for some subgroup analyses despite *a priori* sample size estimation. Therefore, non-significant findings should be interpreted cautiously and regarded as preliminary.

Several methodological constraints should also be acknowledged. First, participants were classified based on HbA1c and FBG without oral glucose tolerance testing, which may have led to misclassification of individuals with isolated impaired glucose tolerance and reduced sensitivity of metabolic phenotyping. Second, tMMP-8 rather than the biologically active form (aMMP-8) was measured, which may limit comparability with studies demonstrating stronger diagnostic performance of aMMP-8 in periodontal disease. Third, the absence of a T2DM comparison group prevents evaluation of biomarker trajectories across the full glycemic spectrum.

In addition, the ELISA assays used were validated for serum/plasma rather than saliva, introducing potential matrix effects; therefore, absolute concentrations should be interpreted as semi-quantitative, while internal group comparisons remain valid. The lack of insulin resistance measures (e.g., fasting insulin or HOMA-IR) further limits mechanistic interpretation, particularly for adiponectin and resistin. Finally, the single-center design in a non-obese (BMI ≤ 30 kg/m²) Indian cohort and the absence of menopausal status data restrict generalisability and introduce unmeasured biological variability.

The cross-sectional design also limits inference about causal relationships between salivary biomarkers and periodontal or metabolic parameters. Future longitudinal studies are warranted to evaluate the predictive value of these biomarkers over time, and incorporating aMMP-8 lateral-flow testing, HOMA-IR, OGTT-based classification, and multi-center design could further enhance the clinical utility of salivary biomarker panels for early detection and monitoring in metabolically at-risk populations.

## 5 Conclusions

Salivary tMMP-8 and adiponectin levels distinguished prediabetic adults with and without periodontitis from systemically and periodontally healthy controls. A PCA-derived composite Inflammatory Profile Score, dominated by elevated tMMP-8 and reduced adiponectin, outperformed single biomarkers in discriminating PreDM-PD from healthy controls (AUC = 0.925), with robust performance after adjustment for age. These findings support the use of multi-marker salivary panels for non-invasive screening of periodontal and metabolic risk in adults with prediabetes and underscore the need for prospective studies that incorporate aMMP-8, OGTT-based classification, and direct measures of insulin resistance.

## Data Availability

The raw data supporting the conclusions of this article will be made available by the authors, without undue reservation.

## Appendix 1

Patient demographics, personal details, oral hygiene status questionnaire

1. Gender (Tick the appropriate answer)

Male Female

2. Age
3. Where is your place of stay? (Tick the appropriate answer)

Rural-urban

4. Height (cm)
5. Weight (kg)
6. Do you suffer from any systemic illness? (Tick the appropriate answer)

No Yes specify

7. Do you take any medication regularly for the systemic illness?

No Yes specify

8. What is your highest educational qualification? (Tick the appropriate answer)

No educational qualifications

Vocational qualification

Degree (e.g., BA, BSc)

Postgraduate qualification

Professional qualification

9. Do you have the habit of smoking? (Tick the appropriate answer)

No Yes occasional

10. Are you employed? No Yes

If yes (Tick the appropriate answer)

- Employed Full-Time
- Employed Part-Time
- Seeking opportunities
- Retired
- Prefer not to say

**Table:**

1. How many times do you brush your teeth?	No brushing.	Once a day.	Twice a day or more.
2. How do you clean your teeth?	Toothbrush, fluoride toothpaste & dental floss.	Toothbrush, fluoride toothpaste.	Toothbrush only.
3. How often do you change your toothbrush?	Once in 3 months.	Once every 6 months or more.	
4. Do you use mouthwashes containing fluoride?	Often.	Sometimes.	Rare or never.
5. Do you complain of halitosis (bad smell from your mouth)?	Often.	Sometimes.	Rare or never.
6. Do you complain of bleeding on brushing or gingival bleeding?	Often.	Sometimes.	Rare or never.
7. How often do you visit the dental clinic for a check-up?	Once a year or more often.	Once every few years or when there is pain.	
8. What procedures do you do the most?	Scaling	Restorations	Extraction
9. How often do you get your teeth cleaned by a dentist?	Rare or never	Once a year	Twice in a year
