# Supplementary material for: Salivary biomarker profiling in prediabetes-associated periodontitis: role of adiponectin, resistin, and total matrix metalloproteinase-8
Source: Front Dent Med. 2026 Jun 19;7:1873996. doi: 10.3389/fdmed.2026.1873996 (PMC13328464; doi:10.3389/fdmed.2026.1873996)
Supplement: Supplementary file 1 [file Table1.docx]

***Supplementary Table 1***

**Demographic Characteristics, oral habits, oral complaints among the groups**

| **Variables** | | **PreDM-PD (n=24)** | **PreDM-PH (n=19)** | **SH-PD**  **(n=22)** | **SH-PH Control (n=19)** | **p-value** |
| --- | --- | --- | --- | --- | --- | --- |
| Age (years) | | 45.58 ± 8.94^a^ | 38.32 ± 7.36^b^ | 46.50 ± 6.12^ac^ | 30.89 ± 10.40^d^ | 0.000* |
| Gender | Male, n (%) | 11 (45.8) | 11 (57.9) | 9 (40.9) | 4 (21.1) | 0.135 |
|  | Female, n (%) | 13 (54.2) | 8 (42.1) | 13 (59.1) | 15 (78.9) | 0.121 |
| place of stay | Rural, n (%) | 6 (25.0) | 12 (63.2) | 9 (40.9) | 6 (31.6) | 0.069 |
|  | Urban, n (%) | 18 (75.0) | 7 (36.8) | 13 (59.1) | 13 (68.4) | 0.069 |
| Smokers | Former, n (%) | 14 (58.3) | 15 (78.9) | 17 (77.3) | 15 (78.9) | 0.325 |
|  | Never, n (%) | 10 (41.7) | 4 (21.1) | 5 (22.7) | 4 (21.1) | 0.345 |
| Frequency of brushing | Once a day, n (%) | 18 (75.0) | 16 (84.2) | 16 (72.7) | 11 (57.9) | 0.331 |
|  | Twice a day, n (%) | 6 (25.0) | 3 (15.8) | 6 (27.3) | 8 (42.1) | 0.333 |
| Flossing habit | Once/twice a day, n (%) | 8 (33.3) | 0 (0.0) | 1 (4.5) | 0 (0.0) | 0.005* |
|  | Occasional, n (%) | 11 (45.8) | 12 (63.2) | 15 (68.2) | 13 (68.4) |  |
|  | Never, n (%) | 5 (20.8) | 7 (36.8) | 6 (27.3) | 6 (31.6) |  |
| Complaints of halitosis | Often, n (%) | 6 (25.0) | 6 (31.6) | 6 (27.3) | 1 (5.3) | 0.121 |
|  | Occasional, n (%) | 12 (41.7) | 4 (21.1) | 8 (36.4) | 6 (31.6) |  |
|  | Never, n (%) | 6 (25.0) | 9 (47.4) | 8 (36.4) | 12 (63.2) |  |
| Bleeding on brushing | Often, n (%) | 8 (33.3) | 6 (31.6) | 7 (31.8) | 1(5.3) | 0.003* |
|  | Occasional, n (%) | 10 (41.7) | 7 (36.8) | 9 (40.5) | 2 (10.5) |  |
|  | Never, n (%) | 6 (25.0) | 6 (31.6) | 6 (27.3) | 16 (84.2) |  |
| How often do you have dental check-up? | Once in a year, n (%) | 10(41.7) | 9(47.4) | 11(50.0) | 9(47.4) | 0.951 |
|  | Occasional/ never, n (%) | 14 (58.3) | 10 (52.6) | 11(50.0) | 10 (52.6) |  |
| Family history of Diabetes | Yes, n (%) | 17(70.8) | 11(57.9) | 13 (59.1) | 8 (42.1) | 0.307 |
|  | No, n (%) | 7 (29.2) | 8 (42.1) | 9 (40.9) | 11(57.9) |  |

**Statistically significant at 1% level (*p*<0.01). Values are expressed as mean ± standard deviation and number (percentage). One-way analysis of variance (ANOVA) followed by post-hoc Tukey test comparisons was used for continuous variables and Pearson’s chi-square test for categorical variables to analyze significant differences. Values with different superscripted letters indicate a statistically significant pairwise difference (*p*<0.05) by Scheffe’s post-hoc test. Values with the same superscripted letters indicate a not statistically significant pairwise difference (*p*>0.05).

Descriptive and categorical variables are expressed as frequency (percentage). *P-values* were tested using Pearson's chi-square test for categorical variables.
