# Supplementary material for: Salivary biomarker profiling in prediabetes-associated periodontitis: role of adiponectin, resistin, and total matrix metalloproteinase-8
Source: Front Dent Med. 2026 Jun 19;7:1873996. doi: 10.3389/fdmed.2026.1873996 (PMC13328464; doi:10.3389/fdmed.2026.1873996)
Supplement: Supplementary file 2 [file Table2.docx]

***Supplementary Table 2***

**Correlation Analysis Between Systemic, Periodontal Parameters and Salivary Biomarkers**

| **Parameters**  **(N=84)** | **MMP-8 (ng/mL)** | **Adiponectin (ng/ml)** | **Resistin (ng/ml)** |
| --- | --- | --- | --- |
| **BMI (kg/m2)** | 0.402^**^ | -0.136 | 0.193 |
| **WC (cm)** | 0.259^*^ | -0.259^*^ | 0.040 |
| **FBS (mg/dl)** | 0.267^*^ | -0.464^**^ | -0.047 |
| **HBA1c (%)** | 0.309^**^ | -0.494^**^ | 0.026 |
| **BoP (%)** | 0.176 | -0.259^*^ | 0.056 |
| **PI (%)** | 0.051 | -0.222^*^ | 0.007 |
| **PPD (mm)** | 0.273^*^ | -0.298^**^ | -0.024 |
| **Recession (mm)** | 0.127 | -0.251^*^ | 0.096 |
| **CAL (mm)** | 0.332^**^ | -0.292^**^ | 0.226^*^ |
| **Number of missing teeth (n)** | 0.096 | -0.092 | 0.062 |

**Statistically significant at 1% level (p<0.01); *Significant at 5% level (p<0.05)

Abbreviations:  BMI, Body mass index; WC, Waist circumference; FBS, Fasting blood sugar; HbA1c, Glycated hemoglobin; BoP, Bleeding on probing; PI, Plaque index; PPD, Probing pocket depth; GR, Gingival recession; CAL, Clinical attachment loss; MMP**-**8, matrix metalloproteinase**-**8
