# Supplementary material for: Salivary biomarker profiling in prediabetes-associated periodontitis: role of adiponectin, resistin, and total matrix metalloproteinase-8
Source: Front Dent Med. 2026 Jun 19;7:1873996. doi: 10.3389/fdmed.2026.1873996 (PMC13328464; doi:10.3389/fdmed.2026.1873996)
Supplement: Supplementary file 3 [file Table3.docx]

***Supplementary Table 3.***

**Component Loadings of Salivary Biomarkers on the Inflammatory Biomarker Profile**

| **Variable** | **Communality** | **Inflammatory Biomarker Profile** |
| --- | --- | --- |
| **MMP-8 (ng/mL)** | 0.771 | 0.861* |
| **Adiponectin (ng/mL)** | 0.613 | -0.603 |
| **Resistin (ng/mL)** | 0.808 | 0.085 |
| **Eigenvalue** |  | 1.177 |
| **% of Variance** |  | 37.10 |

Principal component analysis with Varimax rotation. Inflammatory signature characterized by strong positive loadings for MMP-8 and an inverse loading for Adiponectin.

*Indicate the highest loading for each variable.
